# Circulating MicroRNAs in Plasma Decrease in Response to Sarcopenia in the Elderly

**DOI:** 10.3389/fgene.2020.00167

**Published:** 2020-03-05

**Authors:** Nana He, Yue Lin Zhang, Yue Zhang, Beili Feng, Zaixing Zheng, Dongjuan Wang, Shun Zhang, Qi Guo, Honghua Ye

**Affiliations:** ^1^Department of Experimental Medical Science, HwaMei Hospital, University of Chinese Academy of Sciences, Ningbo, China; ^2^Key Laboratory of Diagnosis and Treatment of Digestive System Tumors of Zhejiang Province, Ningbo, China; ^3^Department of Cardiology, HwaMei Hospital, University of Chinese Academy of Sciences, Ningbo, China; ^4^Shanghai University of Medicine and Health Sciences Affiliated Zhoupu Hospital, Shanghai, China

**Keywords:** circulating microRNAs, sarcopenia, biomarker, plasma, elderly

## Abstract

sarcopenia has been defined as the aging-related disease with the declined mass, strength, and function of skeletal muscle, which is a major cause of morbidity and mortality in elders. Current diagnostic criteria of sarcopenia have not been agreed internationally, and the clinical diagnostic biomarkers for sarcopenia have not been identified. Circulating miRNAs (miRNAs, miRs) have recently been characterized as novel biomarkers for sarcopenia. However, the change of circulating miRNAs in response to sarcopenia are still not fully understood. Here, we enrolled a total of 93 elderly patients clinically diagnosed with sarcopenia and matching 93 non-sarcopenia elderly in this study. Specifically, levels of candidate circulating miRNAs which were involved in angiogenesis, inflammation and enriched in muscle and/or cardiac tissues were detected in these two groups. In small-sample screening experiments, plasma miR-155, miR-208b, miR-222, miR-210, miR-328, and miR-499 levels were significantly down-regulated in sarcopenia compared to those who non-sarcopenia. In contrast, miR-1, mir-133a, miR-133b, miR-21, miR-146a, miR-126, miR-221, and miR-20a were not changed significantly. Subsequently, we expanded the sample size to further detection and verification, and found that plasma miR-155, miR-208b, miR-222, miR-210, miR-328, and miR-499 levels in the sarcopenia group were significantly reduced compared to the non-sarcoma group, which is consistent with the results of the small-sample screening experiment. In addition, we showed that ASM/Height2, handgrip strength, knee extension and 4-meter velocity in sarcopenia group were significantly lower than those in non-sarcopenia group. Here we correlated the decrease of miR-208b, miR-499, miR-155, miR-222, miR-328, and miR-210 in sarcopenia group and non-sarcopenia group with diagnostic indexes of sarcopenia (ASM/Height2, Handgrip strength and 4-meter velocity) after adjusting sex. The results showed that miR-208b and miR-155 changes were significantly correlated with handgrip strength in woman, miR-208b, miR-499, and miR-222 changes were significantly correlated with ASM/Height2 in man, while other miRNAs changes did not show a strong correlation with these diagnostic indexes. In conclusion, plasma miR-208b, miR-499, miR-155, miR-222, miR-328, and miR-210 decrease in response to sarcopenia in the elderly. Although further studies are needed to clarify the potential use of circulating miRNAs as biomarkers of sarcopenia, present findings set the stage for defining circulating miRNAs as biomarkers and suggesting their physiological roles in elderly with sarcopenia.

## Introduction

With the increasing pressure of global aging population, diseases related to the elderly are of great concern. As a major cause of death and disability in the elderly, sarcopenia has been widely recognized and concerned. Sarcopenia is a syndrome characterized by the decline of skeletal muscle mass, a progressive loss of muscle strength and function with age (Chen et al., 2014; Landi et al., 2018; Sanchez-Rodriguez et al., 2019). The occurrence of sarcopenia is a complex process, which is controlled by both extrinsic and intrinsic factors, including the decrease of exercise volume, the change of hormone level, the degeneration of motor neurons and the metabolic disorder of nutritional factors (Cruz-Jentoft et al., 2019). Recently, there have been more and more studies on sarcopenia, but most of them are still in the exploratory stage, and the pathogenesis and diagnostic criteria are still unclear.

Due to the increase of age, the body’s metabolic capacity is gradually decreasing, and the incidence of sarcopenia has also increased (Cruz-Jentoft et al., 2014). It is easy to trigger a series of health problems. Sarcopenia is associated with increased adverse outcomes including falls, functional decline, frailty, and mortality (Bjorkman et al., 2019; Yang et al., 2019). Sarcopenia also reduces the amount of metabolically active tissue; thus, it increases the risk for metabolic diseases, including cardiovascular disease, diabetes, hypertension and hyperlipidemia and other elderly diseases (Biolo et al., 2014; Wang et al., 2018; Yilmaz and Bahat, 2019). This will bring heavy economic burden to the family of the elderly, society and medical service system. However, the harm caused by sarcopenia has not yet attracted enough attention from clinicians and society.

MicroRNA (miRNA, miR) is regarded as the most promising small molecule RNA for targeted diagnosis and treatment in the 21st century, which plays a key role in a wide range of physiological and pathological processes (Brown and Goljanek-Whysall, 2015). MiRNAs are a class of non-coding regulatory RNA molecules with a length of about 20–30 nucleotides that regulate gene expression at the post-transcriptional level. Some miRNAs are ubiquitously expressed in tissue, while others are tissue-specific or tissue-enriched (e.g., skeletal muscle). MiRNAs can be released by cells and are found in various body fluid, including serum and plasma (Gautam et al., 2016). Therefore, detecting changes in miRNA levels in the circulation can provide information about the original tissue or cell. At present, people are mainly concerned about the function of miRNA in tissues and cells. Increasing evidence has reported that circulating miRNAs are present in serum or plasma, and circulating miRNAs are remarkably stable, easily detectable, and sensitive to changes in health status (Kroh et al., 2010; Jung and Suh, 2014; Troiano et al., 2016). Therefore, miRNAs in serum or plasma can be used as potential biomarkers for the diagnosis and treatment of diseases. To date, a growing number of evidence suggests that circulating miRNAs as potential therapeutic biomarkers for sarcopenia, and a number of circulating miRNAs are found to be associated with age-related muscle missing in elderly (McGregor et al., 2014; Siracusa et al., 2018). Recently, several works have demonstrated that various circulating miRNAs are differentially expressed during aging. However, the response of circulating miRNAs to sarcopenia in elderly is undetermined.

Here, we investigate how specific circulating miRNAs with well-established roles is linked to sarcopenia in elderly. Specifically, we determined the change of circulating miRNA levels in elderly with sarcopenia and non- sarcopenia. We fund that plasma miR-208b, miR-499, miR-155, miR-222, miR-328, and miR-210 levels were significantly down-regulated in response to sarcopenia in elderly, while miR-1, mir-133a, miR-133b, miR-21, miR-146a, miR-126, miR-221, and miR-20a were not changed significantly. Moreover, we correlated the circulating miRNA changes with diagnostic indicators of sarcopenia (ASM/Height2, Handgrip strength and 4-meter velocity), showing the potential of circulating miRNA as biomarkers of sarcopenia in elderly.

## Materials and Methods

### Participants

All subjects in this study were from Ximen Community of Ningbo, China. A total of 1047 older individuals (age ≥65 years) were enrolled our examination program and finished a comprehensive geriatric assessment from November 2016 to March 2017. Inclusion criteria of this trial: (1) older individuals (age ≥65 years); and (2) People who can independently detect walking speed, grip strength and muscle mass. Exclusion criteria of this trial: (1) People who refused to participate in the study; (2) People who cannot complete the required inspection items independently; and (3) older individuals (age ≤65 years). According to the AWGs criteria for the diagnosis of sarcopenia, the subjects were further divided into sarcopenia group (*n* = 93) and non-sarcopenia group (*n* = 93). The matching factors were gender and age. We adhered to the principles of the Declaration of Helsinki, and the study protocol was approved by the Ethics Committee of Ningbo No. 2 Hospital. All participants gave informed written consent.

### Plasma Sampling

Venous blood was collected in silicone-coated serum tubes with increased silica act clot activator, followed by processing within 1 h after collection. At 4°C, blood samples were centrifuged at 3,000 rpm for 15 min, and plasma and erythrocytes were separated. Aliquots of plasma were collected into RNase/DNase-free tubes and immediately aliquoted and frozen at −80°C until the assays were performed.

### RNA Isolation

The total RNA extraction was performed using a mirVana PARIS isolation kit (Ambion, Austin, Texas) according to the manufacturer’s instructions. To avoid variability of results, repeated freeze-thaw cycles of serum samples were minimized and all samples were extracted and analyzed in a single batch. Briefly, 400 μl of plasma was used to extract the total RNA. After equal volume of denaturing solution was added, 50 pmol/L Caenorhabditis elegans miR-39 (cel-miR-39) was added to normalize the miRNA serum levels.

### Quantification of Circulating MiRNA Levels

For quantitative miRNA analysis, Bulge-LoopTM miRNA qPCR Primer Sets (RiboBio) were used to detect selected miRNAs expressions by quantitative reverse transcription polymerase chain reactions (qRT-PCRs) with iTaqTM Universal SYBR Green Supermix (BIO-RAD) in the 7900HT Fast Real-Time PCR System as previously reported. All qRT-PCR reactions were performed in triplicate, and the signal was collected at the end of every cycle. As there is no consensus on endogenous stable miRNAs in the circulation to act as house-keepers, the expression level of miRNAs in serum were normalized using spike-in cel-miR-39, which lacks sequence homology to human miRNAs.

### Assessment of Muscle Strength and Physical Performance

Body composition features were measured by a direct segmental multifrequency bioelectrical impedance analysis; Appendicular skeletal muscle mass (ASM) was calculated as the sum of skeletal muscle in the arms and legs; Relative skeletal muscle mass index (ASM/Ht2) was defined as ASM divided by body height in meters squared; We collected muscle strength to the nearest 0.1 kg with a accurate handgrip dynamometer; The 4-meter walking speed test was carried out on a straight corridor with a 6-meter mark on the ground.

### Other Measurement

Each participant’s age, gender, occupation, medical history, intake of medications, and smoking and drinking habits were asked by an experienced staff through a standardized questionnaire. They measured body height and waist circumference to the nearest 0.5 cm. Body mass index was weight in kilograms divided by the height in meters squared.

Office blood pressure was measured by means of the Omron HEM-1300 monitor (Omron Healthcare, Inc., Kyoto, Japan). After participants had rested in the sitting position for at least 5 min 3 consecutive blood pressure readings were obtained according to the recommendations of the European Society of Hypertension. For analysis, the three readings were averaged. Office hypertension was a blood pressure of at least 140 mmHg systolic or 90 mmHg diastolic.

Venous blood samples, obtained after overnight fasting, were analyzed by automated enzymatic methods for serum total and high-density lipoprotein cholesterol, serum creatinine and uric acid, and plasma glucose. Hypertension was a former diagnosis or use of antihypertensive drugs. Diabetes mellitus was a glycosylated hemoglobin level of 7.0% or higher or use of anti-diabetic drugs, or prior diagnosis. Dyslipidaemia was a total cholesterol concentration above 5.0 mmol/L, a high-density-lipoprotein cholesterol level lower than 1.2 mmol/L in women or below 1.0 mmol/L in men, or use of lipid-lowering drugs.

### Statistical Analysis

Subject characteristics, biochemical measurements and general echocardiographic indexes are presented as means ± standard error of the mean (SEM). For the analysis of qRT-PCR data, the relative expression level for each miRNA was calculated using the 2 ^–ΔΔCt^ method and the data were expressed as the mean ± SE. Paired-samples were compared by *t*-test as appropriate for analyzing the changes of miRNAs. Correlation analyses between changes of circulating miRNAs and diagnostic indexes of sarcopenia (ASM/Height2, Handgrip strength and 4-meter velocity) were performed using the Pearson’s method as appropriate for data distribution. Statistical significance is defined as *P*-values <0.05.

## Results

### Subject Characteristics

The cohort used in this study is the same as previously reported (Zhang et al., 2019). The clinical characteristics for these subjects were shown in Table 1. The height of the non-sarcopenia group is higher than that of the sarcopenia group (159.73 ± 0.78 vs. 156.03 ± 0.74, *p* < *0.05*); Body mass in non-sarcopenia group is greater than that in sarcopenia group (64.72 ± 1.19 vs. 52.97 ± 0.80, *p* < *0.05*); BMI in non-sarcopenia group is greater than that in sarcopenia group (25.32 ± 0.41 vs. 21.75 ± 0.29, *p* < *0.05*). There was no significant difference in age, systolic blood pressure and diastolic blood pressure between the two groups. Table 2 lists the detailed anthropometric indexes of participants. ASM/Height2, handgrip strength, knee extension and 4-meter velocity in sarcopenia group were significantly lower than those in non-sarcopenia group (*p* < *0.05*). There was no significant difference of body muscle mass (%) and body fat mass (%) between the two groups. We analyzed the biochemical measurements of participants in non-sarcopenia and sarcopenia group (Table 3). The results showed that there were no significant changes of blood lipids (Total cholesterol, LDL cholesterol, Triglyceride), renal function (serum creatinine), arterial stiffness (BaPWV) and plasma glucose (HbA1c) between non-sarcopenia and sarcopenia group, while blood lipids marker HDL cholesterol of sarcopenia group was significantly higher than that of non-sarcopenia group.

**TABLE 1:** Clinical characteristic of participants.

	Mean ± SEM		
Clinical	Non-sarcopenia	sarcopenia	*P*
parameters	group (*n* = 93)	group (*n* = 93)	
Age(years)	76.19 ± 0.58	76.15 ± 0.58	>0.05
Height(cm)	159.73 ± 0.78	156.03 ± 0.74	<0.05
Body mass(kg)	64.72 ± 1.19	52.97 ± 0.80	<0.05
BMI(kg/m^2^)	25.32 ± 0.41	21.75 ± 0.29	<0.05
Systolic blood pressure (mmHg)	140.11 ± 1.80	137.35 ± 2.44	>0.05
Diastolic blood pressure (mmHg)	72.18 ± 1.09	72.36 ± 1.30	>0.05
Pressure (mmHg)			

**TABLE 2:** Anthropometric indexes of participants.

	Mean ± SEM		
Anthropometric	Non-sarcopenia	sarcopenia	*p*
indexes	group	group	
ASM/Height^2^(kg/m^2^)	6.71 ± 0.10	5.63 ± 0.07	<0.05
Body muscle mass(%)	62.64 ± 0.67	64.23 ± 0.71	>0.05
Body fat mass(%)	33.55 ± 0.72	31.71 ± 0.75	>0.05
Handgrip strength(kg)	24.18 ± 0.94	17.33 ± 0.55	<0.05
Knee extension(kg)	19.93 ± 0.81	14.76 ± 0.59	<0.05
4-meter velocity(m/s)	1.12 ± 0.02	1.00 ± 0.03	<0.05

**TABLE 3:** Physiological functions of participants.

	Mean ± SEM		
Physiological	Non-sarcopenia	sarcopenia	*p*
functions	group	group	
**Blood lipids**			
Total cholesterol(mmol/L)	4.42 ± 0.11	4.69 ± 0.11	>0.05
HDL cholesterol(mmol/L)	1.48 ± 0.04	1.69 ± 0.05	<0.05
LDL cholesterol(mmol/L)	2.84 ± 0.10	3.04 ± 0.10	>0.05
Triglyceride(mmol/L)	1.69 ± 0.10	1.44 ± 0.07	>0.05
**Renal function**			
Serum creatinine(μmol/L)	75.01 ± 2.31	74.42 ± 2.98	>0.05
Arterial stiffness			
BaPWV(cm/s)	1883.14 ± 41.45	1999.18 ± 50.76	>0.05
**Plasma glucose**			
HbA1c(%)	6.22 ± 0.09	6.17 ± 0.10	>0.05

### Circulating MiR-155, MiR-208b, MiR-222, MiR-210, MiR-328, and MiR-499 Decrease in Response to Sarcopenia in the Elderly

**FIGURE 1:**
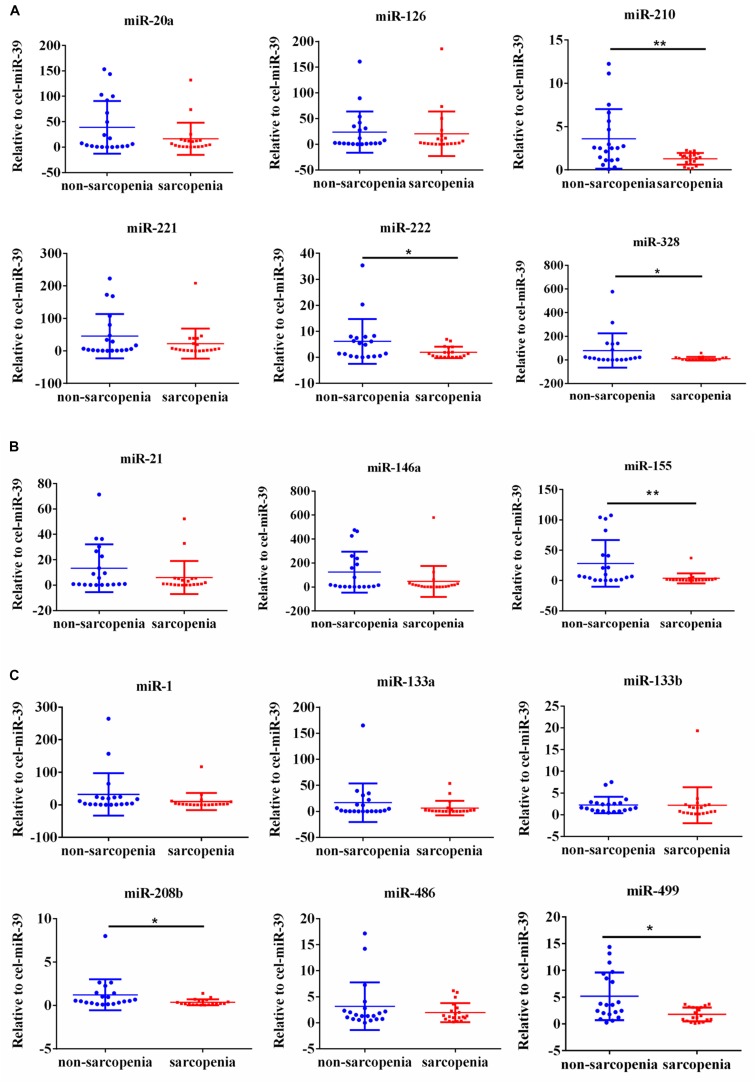
Changes in circulating microRNAs in response to sarcopenia in the elderly. **(A)** Changes of angiogenesis-related miRNAs when normalized to cel-miR-39 in small-sample screening experiment; *compared to non-sarcopenia of the elderly, *P* < *0.05*; **compared to non-sarcopenia of the elderly, *P*< *0.01*. **(B)** Changes of inflammation-related miRNAs when normalized to cel-miR-39 in small-sample screening experiment; *compared to non-sarcopenia of the elderly, *P* < *0.05*; **compared to non-sarcopenia of the elderly, *P*< *0.01*. **(C)** Changes of cardiac or muscle-specific/enriched miRNAs when normalized to cel-miR-39 in small-sample screening experiment; *compared to non-sarcopenia of the elderly, *P* < *0.05*; **compared to non-sarcopenia of the elderly, *P*< *0.01*.

**FIGURE 2:**
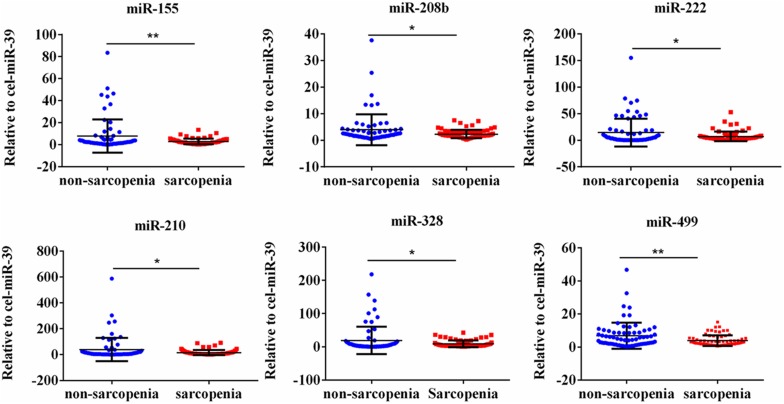
CirculatingmiR-155, miR-208b, miR-222, miR-210, miR-328 and miR-499 decrease in response to age-associated loss of muscle in elderly patients; *compared to non-sarcopenia of the elderly, *P* < *0.05*; **compared to non-sarcopenia of the elderly, *P*< *0.01*.

We determined the expression of angiogenesis-related miRNAs (miR-20a, miR-126, miR-210, miR-221, miR-222, and miR-328) (Dews et al., 2006; Poliseno et al., 2006; Kuehbacher et al., 2007; Fasanaro et al., 2008; Soeki et al., 2016), inflammation-related miRNAs (miR-21, miR-146a, and miR-155) (Taganov et al., 2006; Urbich et al., 2008; Wang et al., 2017) and cardiac or muscle-specific/enriched miRNAs (miR-1, miR-133a, miR-133b, miR-208b, miR-486, and miR-499) (Chen et al., 2006; Alexander et al., 2014; Soci et al., 2016). Firstly, the results of small-sample screening experiment showed that plasma miR-208b, miR-499, miR-155, miR-222, miR-328, and miR-210 levels were significantly down-regulated in sarcopenia group compared to those who non-sarcopenia. In contrast, miR-1, mir-133a, miR-133b, miR-21, miR-146a, miR-126, miR-221, and miR-20a were not changed significantly (Figure 1). Then, we further expanded the experimental sample size to verify the expression trend of these differentially expressed miRNAs. The results also showed that miR-208b, miR-499, miR-155, miR-222, miR-328, and miR-210 levels were significantly down-regulated in sarcopenia group compared to those who non-sarcopenia (Figure 2).

### Correlation Analysis Between Changes of MiR-208b, MiR-499, MiR-155, MiR-222, MiR-328 and MiR-210 and ASM/Height2, Handgrip Strength or 4-Meter Velocity

**FIGURE 3:**
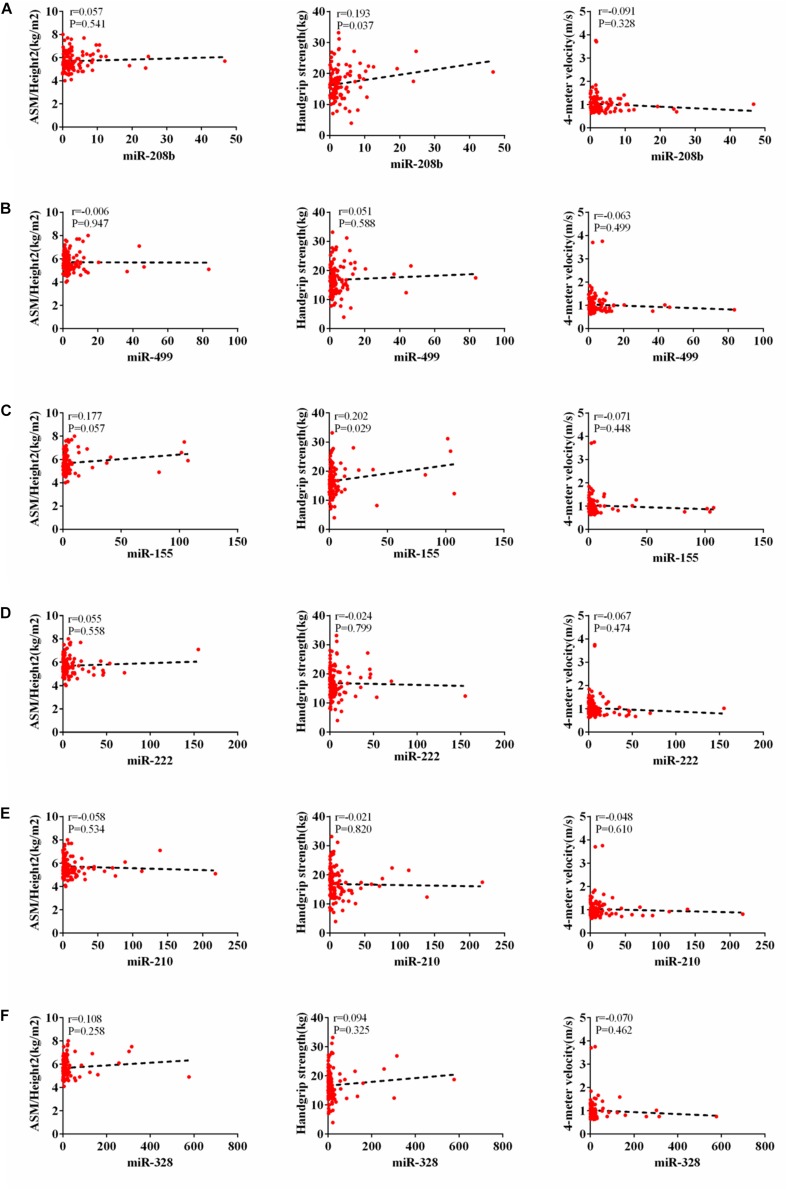
Correlation analysis between the changes of miR-208b **(A)**, miR-499 **(B)**, miR-155 **(C)**, miR-222 **(D)**, miR-210 **(E)** and miR-328 **(F)** and ASM/Height2(kg/m2), Handgrip strength(kg) and 4-meter velocity(m/s) in woman, *P* < *0.05*.

**FIGURE 4:**
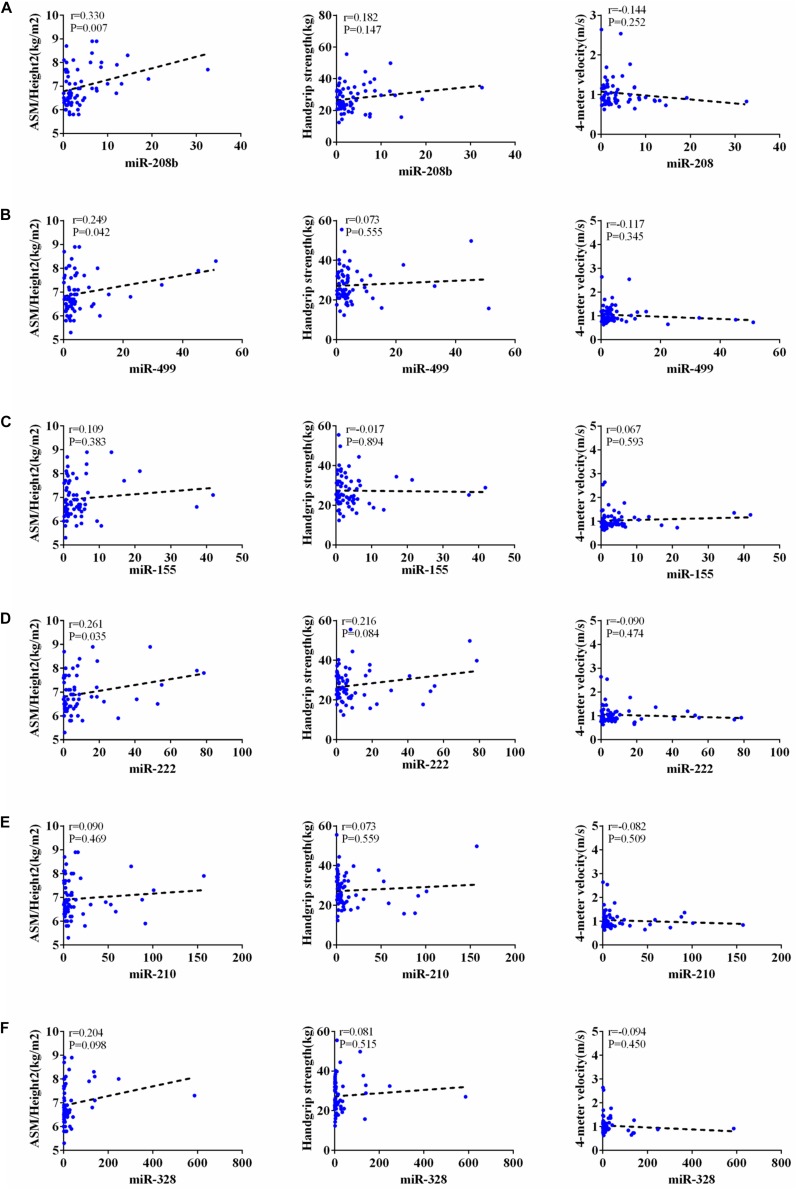
Correlation analysis between the changes of miR-208b **(A)**, miR-499 **(B)**, miR-155 **(C)**, miR-222 **(D)**, miR-210 **(E)** and miR-328 **(F)** and ASM/Height2(kg/m2), Handgrip strength(kg) and 4-meter velocity(m/s) in man, *P* < *0.05*.

According to AWGs standard, sarcopenia was diagnosed as low muscle mass, low muscle strength and/or low physical strength. ASM/height^2^, handgrip strength and 4- meter velocity were used for detection respectively. The levels of ASM/height^2^, handgrip strength and knee extension were significantly decreased in the elderly with sarcopenia (Table 2). Here we correlated the decrease of miR-208b, miR-499, miR-155, miR-222, miR-328, and miR-210 in sarcopenia group and non-sarcopenia group with diagnostic indicators of sarcopenia (ASM/Height^2^, Handgrip strength and 4-meter velocity) after adjusting sex. The results showed that miR-208b and miR-155 changes were significantly correlated with handgrip strength in woman (Figure 3), miR-208b, miR-499, and miR-222 changes were significantly correlated with ASM/Height2 in man (Figure 4), while other miRNAs changes did not show a strong correlation with these diagnostic indexes.

## Discussion

Sarcopenia is a progressive decline in skeletal muscle mass, strength, and quality during aging. Although the molecular mechanisms involved in sarcopenia development are not entirely understood, it is well-documented that transcriptomic, proteomic and epigenetic changes during the progression of aging are the main causative factors for sarcopenia. This study was designed to explore how specific circulating miRNAs are changed in response to sarcopenia in the elderly. Here we reported that plasma miR-208b, miR-499, miR-155, miR-222, miR-328, and miR-210 levels were significantly down-regulated in sarcopenia compared to those who non-sarcopenia. We also showed that the levels of ASM/height2, handgrip strength, knee extension and 4-meter velocity were significantly decreased in elderly with sarcopenia. Here we correlated the decrease of miR-208b, miR-499, miR-155, miR-222, miR-328, and miR-210 with predictor of sarcopenia (ASM/Height2, Handgrip strength and 4-meter velocity) after adjusting sex.

The results showed that miR-208b and miR-155 changes were significantly correlated with handgrip strength in woman, miR-208b, miR-499, and miR-222 changes were significantly correlated with ASM/Height2 in man, while other miRNAs changes did not show a strong correlation with these diagnostic indexes. Given that miRNA changes were correlated with diagnostic indicators of sarcopenia, the present study also provides potential biomarkers for sarcopenia in the elderly. Moreover, the potential biological function of these dysregulated miRNAs responsible for beneficial effects of sarcopenia in elderly warrants further studied.

Several circulating miRNAs have been identified as closely related to sarcopenia in the elderly. Increasing evidence has shown that miRNAs are differentially expressed in skeletal muscle with age. Specifically, miR-1, miR-21, mir-133a, miR-133b, miR-206, miR-146a, and miR-155 were up-regulated while miR-20a, miR-208b, miR-210, miR-221, miR-222, and miR-499 were down-regulated in aged muscle compared to young muscle (Kim et al., 2014; Margolis and Rivas, 2018; Jung et al., 2019). However, all these studies were conducted in aged muscle. To the best of our knowledge, the present study is the first to identify plasma miRNAs that change in response to sarcopenia in the elderly. The differentially expressed miRNAs in earlier studies appeared consistently in the plasma of sarcopenia in the elderly from our study. For example, the expression pattern of miR-208b, miR-499, miR-222, and miR-210 are similar to previous studies. Here we reported that plasma miR-208b, miR-499, miR-222, and miR-210 were significantly down-regulated in plasma in the elderly with sarcopenia while other miRNAs determined in this study were not changed, indicating that individual differences of participants might affect levels of circulating miRNAs. Considering most of the previous studies focused on miRNA expression changes in aged muscles, our results of circulating miRNA expression in plasma provide new insights for the diagnosis and treatment of sarcopenia.

Sarcopenia is a kind of senile syndrome, and its pathogenesis is very complex. Many diseases can cause muscle changes, resulting in decreased muscle mass and strength, so there is a close relationship between sarcopenia and various diseases. According to statistics from the literature, the incidence of sarcopenia in patients with liver cirrhosis is 40 to 70%, which is much higher than the incidence of sarcopenia in patients without liver disease (Dasarathy, 2012; Sinclair et al., 2016). At the same time, sarcopenia is one of the most common complications in patients with cirrhosis, and there are few effective treatments for it. Studies have shown that sarcopenia is one of the risk factors for chronic cardiovascular disease. Patients with heart failure (HF) often suffer from sarcopenia at the same time, and improving muscle loss in HF patients is an effective treatment strategy (Chin et al., 2013; Loncar et al., 2013; Cipryan, 2018). It was reported that the muscle mass, strength and function of the leg muscles of diabetic patients are rapidly decreasing compared with those with normal blood glucose, and it has been proposed that the development of personalized functional exercises for the elderly with diabetes is of great significance in preventing and treating sarcopenia (Leenders et al., 2013; Wang et al., 2018). The miRNAs selected in this study have potential biological relevance in sarcopenia-related diseases, including diabetes, cardiovascular disease, and inflammation. Among the miRNAs analyzed here are angiogenesis-related miRNAs (miR-20a, miR-126, miR-210, miR-221, miR-222, and miR-328), inflammation-related miRNAs (miR-21, miR-146a, and miR-155) and cardiac or muscle-specific/enriched miRNAs (miR-1, miR-133a, miR-133b, miR-208b, miR-486, and miR-499). Among them, miR-208b, miR-499, miR-155, miR-222, and miR-210 were significantly down-regulated in the plasma of sarcopenia in the elderly. Here, our results indicate that these miRNAs may be promising biomarkers for sarcopenia, and have important significance for the screening of early sarcopenia and improvement of sarcopenia-related diseases.

Because the current diagnostic criteria for sarcopenia use ASM/Height2, Handgrip strength and 4-meter velocity, these diagnostic indicators can indicate the severity of sarcopenia (Chen et al., 2014). According to the consensus report of the Asian Working Group on sarcopenia (AWGS) in 2014, we found that the diagnostic criteria for sarcopenia are different between men and women: muscle mass assessment using ASM/Height2, ASM/Height2 ≤ 7.0 kg/m2 (male), ASM/Height2 ≤ 5.7 kg/m2 (female); the handgrip strength was used for the measurement of muscle strength. The cut point of muscle strength was less than 26 kg for men and less than 18 kg for women; 4-meter velocity is selected for muscle function measurement, if 4-meter velocity ≤0.8 m/s, indicates a decline in motor ability. In this study, we correlated the decrease of miR-208b, miR-499, miR-155, miR-222, miR-328, and miR-210 with diagnostic indexes of sarcopenia (ASM/Height2, Handgrip strength and 4-meter velocity) after adjusting sex.

The results showed that miR-208b and miR-155 changes were significantly correlated with handgrip strength in woman, miR-208b, miR-499, and miR-222 changes were significantly correlated with ASM/Height2 in man, while other miRNAs changes did not show a strong correlation with these diagnostic indexes. The results of this study suggest that with the decrease of ASM/hight2, handgrip strength and 4-meter velocity in patients with sarcopenia, the level of miRNA in plasma is gradually reduced. The detection of the level of miRNAs in plasma is helpful for the diagnosis of sarcopenia, indicating that circulating miRNAs can be used as a potential biomarkers of sarcopenia.

As an initial study, several limitations of this study were still existent. On the one hand, the sample size is limited, and the sample source is regional in this study, It is still necessary to increase the sample size, expand the sample coverage area and include more analysis indicators to confirm the application of plasma miRNAs in the diagnosis of sarcopenia. On the other hand, the underlying molecular mechanism between circulating miRNA and sarcopenia is unclear. As more and more miRNAs have been found, their roles in biology have been paid more and more attention. The regulatory network of miRNAs mainly involves post-transcriptional regulation (Kong et al., 2008). However, miRNAs, mRNA and their specific molecular mechanism of disease are still unclear. The regulatory role of miRNAs is very complicated. One miRNA can have hundreds of target genes, and one mRNA may be regulated by multiple miRNAs (Dalmay, 2013). The regulatory mechanism of miRNAs on sarcopenia may be through the regulation of multiple target gene expressions, multiple signal pathways, and they cross each other and interact. In our next study, the molecular mechanisms underlying circulating miRNAs which were related with sarcopenia should be further elucidated.

## Conclusion

In conclusion, plasma miR-208b, miR-499, miR-155, miR-222, miR-328, and miR-210 decrease in response to sarcopenia in the elderly. Future studies aim at providing an opportunity to develop circulating miRNAs as potential and useful biomarkers of sarcopenia and to elucidate the molecular mechanisms underlying muscle dysfunction with age.

## Data Availability Statement

The datasets generated for this study are available on request to the corresponding author.

## Ethics Statement

Ethical approval for this study was obtained from the Ethics Community of Ningbo No. 2 Hospital. All subjects gave written informed consent in accordance with the Declaration of Helsinki.

## Author Contributions

HY and NH conceived and designed this study. NH performed the experiments. NH, YLZ, and YZ collected and analyzed the data. NH, BF, ZZ, DW, SZ, and QG contributed reagents, materials, and analysis tools used in this study. NH wrote the manuscript. HY revised the manuscript.

## Conflict of Interest

The authors declare that the research was conducted in the absence of any commercial or financial relationships that could be construed as a potential conflict of interest.
